# Highly Adhesive Antimicrobial Coatings for External Fixation Devices

**DOI:** 10.3390/gels9080639

**Published:** 2023-08-08

**Authors:** Mikhail Bredikhin, Sushant Sawant, Christopher Gross, Erik L. S. Antonio, Nikolay Borodinov, Igor Luzinov, Alexey Vertegel

**Affiliations:** 1 Department of Bioengineering, Clemson University, Clemson, SC 29634, USA; mbredik@g.clemson.edu (M.B.); sawant@g.clemson.edu (S.S.); 2 Department of Orthopedic Surgery, Medical University of South Carolina, Charleston, SC 29425, USA; grossc@musc.edu; 3 Department of Materials Science and Enfineering, Clemson University, Clemson, SC 29634, USA; asanch2@g.clemson.edu (E.L.S.A.); nikolab@g.clemson.edu (N.B.); luzinov@clemson.edu (I.L.)

**Keywords:** K-wires, antimicrobial gels, chitosan, PGMA, highly adherent, orthopedic implants

## Abstract

Pin site infections arise from the use of percutaneous pinning techniques (as seen in skeletal traction, percutaneous fracture pinning, and external fixation for fracture stabilization or complex deformity reconstruction). These sites are niduses for infection because the skin barrier is disrupted, allowing for bacteria to enter a previously privileged area. After external fixation, the rate of pin site infections can reach up to 100%. Following pin site infection, the pin may loosen, causing increased pain (increasing narcotic usage) and decreasing the fixation of the fracture or deformity correction construct. More serious complications include osteomyelitis and deep tissue infections. Due to the morbidity and costs associated with its sequelae, strategies to reduce pin site infections are vital. Current strategies for preventing implant-associated infections include coatings with antibiotics, antimicrobial polymers and peptides, silver, and other antiseptics like chlorhexidine and silver-sulfadiazine. Problems facing the development of antimicrobial coatings on orthopedic implants and, specifically, on pins known as Kirschner wires (or K-wires) include poor adhesion of the drug-eluting layer, which is easily removed by shear forces during the implantation. Development of highly adhesive drug-eluting coatings could therefore lead to improved antimicrobial efficacy of these devices and ultimately reduce the burden of pin site infections. In response to this need, we developed two types of gel coatings: synthetic poly-glycidyl methacrylate-based and natural-chitosan-based. Upon drying, these gel coatings showed strong adhesion to pins and remained undamaged after the application of strong shear forces. We also demonstrated that antibiotics can be incorporated into these gels, and a K-wire with such a coating retained antimicrobial efficacy after drilling into and removal from a bone. Such a coating could be invaluable for K-wires and other orthopedic implants that experience strong shear forces during their implantation.

## 1. Introduction

The use of implants in orthopedic surgery dates back to the beginning of the 20th century [1]. As surgical techniques and engineering have evolved, orthopedic implants are extremely versatile and can be used by surgeons to replace a missing portion of bone or joint or to support a damaged one. However, since the first applications of synthetic biomaterials in medicine, infections and insufficient tissue integration have been the most frequent and devastating complications [2,3]. Indeed, implant-associated infections comprise one-fourth of all “healthcare-related infections” in the US [4].

External fixation devices are commonly utilized by orthopedic surgeons to treat severely comminuted fractures and complex deformities, as well as for limb lengthening [5,6]. External fixators usually involve the construction of an external frame that is attached to the bone via pins and wires which protrude through the skin and into the bone. This technique is often preferred to internal fixation due to its ability to be applied rapidly, the lack of soft tissue dissection, and the potential for fixation adjustment during bone healing [5]. The metallic wires that are used in external fixation were developed in 1909 by Martin Kirschner and are now called Kirshner wires or pins (K-wires) [7]. A K-wire’s functions include: penetrating and holding bone fragments together, providing an anchor for skeletal traction, and guided cannulated screw placement [7,8,9]. In some instances, they are driven into the bone using a power drill and stay inserted until the fracture heals, which is typically 2–4 months [7].

Complications associated with this procedure vary from minor to life-threatening and can occur immediately after the implantation [10]. For example, heat generation and the subsequent temperature rise caused by the friction between the rotating wire and bone can result in thermal osteonecrosis, which, in turn, gives rise to pin loosening and eventual loss of fixation [7,8,9,11]. Additionally, it can lead to K-wire migration, during which the fixation is lost and the pin deviates from its intended anatomic location [12,13]. Bending the distal end of the K-wire prevents this migration in most cases, but even bent wires can migrate after breakage, so periodic radiographs should be made until the wire is removed [14]. A few strategies have been proposed to decrease the heat generation during the drilling, including tip design, drilling technique, and cooling methods [7,8,9,11,15,16,17]. Hammering, as opposed to rotary drilling, has been one of the more recent trends to reduce the thermal stressing of osteocytes [18].

Nonetheless, pin site infection (PSI) remains the most prominent complication following the external fixation procedure, with the incidence rate up to 100% in some studies [5,6,19,20]. As the demand for these clinical devices continues to grow, the number of these infections rises dramatically along with it, despite the tremendous improvements in perioperative technique [21]. Pin sites are the skin entry points of the skin–metal interface, and PSIs often lead to bone tissue infections, which cause osteomyelitis—the microbial colonization of the bone and its consequential destruction [22]. Unless detected early, these infections can cause bony destruction, instigate systemic sepsis, and even lead to death [23,24,25]. The literature provides conflicting reports on whether burying K-wires under the skin decreases the risk of infection. However, it considerably increases the cost of surgery and prevents quick fixation adjustment during the healing [26,27,28].

Besides the skin penetration at the pin site, which provides an entry for the microbes, the K-wire itself presents a threat in terms of infection. The metal surface acts as an anchor for bacteria to adhere and become embedded in a dense matrix of proteins, polysaccharides, and DNA, forming a biofilm [21]. Bacteria in biofilms are significantly less susceptible to antibiotics than planktonic bacteria [22,29]. Additionally, since neither the host immune response nor prescribed antibiotics are able to eliminate bacteria in biofilms in most cases, a chronic inflammatory response at the biofilm site may be produced [22]. Formation of a biofilm on the K-wire aids in subsequent colonization of the implant and surrounding tissues, and quite often, the only solution is the premature removal of the wire, which can undo surgery and costs billions of dollars’ worth of annual healthcare burden, as well as patients’ suffering [21,24,30].

The current clinical solutions for reducing the risk of PSI include the application of antimicrobial dressings (commonly silver-sulfadiazine- and chlorhexidine-based) on the exposed part of the K-wire and postoperative courses of oral antibiotics [19]. However, there is no consensus on either the regimen or the type of dressing for the appropriate pin care [5,19,21]. One study reported no significant difference in terms of infection for patients with and without daily pin site care [31]. Furthermore, the randomized data from another study suggested that oral antibiotics did not alter the incidence, timing, or severity of PSI [6].

Drug-eluting coatings have been the most prevalent approach to reducing the rate of implant-related infections [5,32]. Most commonly, the drug is loaded into a poly(lactide) or poly(lactide-co-glycolide) coating (PLA or PLGA, respectively), which allows for enhanced drug loading and a regulated drug release rate. Such an approach is utilized in all FDA-approved drug-eluting devices (e.g., C-QUR™ hernia meshes, TAXUS stents, In.Pact^TM^Admiral^TM^ balloons) and serves as the “gold standard” in their further development. Multiple studies have tested antibiotic-eluting coatings of K-wires, including a number of in vivo reports that showed some promising results [2,21,23,24,25,30,33,34,35,36,37,38,39,40,41,42,43]. However, to our best knowledge, there are no peer-reviewed publications in which the tested K-wires were drilled into the cortical compact bone or its analog. For example, in one study, the K-wires were inserted in the pre-drilled holes [37], while in another study, they were drilled into the trabecular cancellous bone [36], which is spongy and much softer than a cortical bone. In all the other studies, drug-eluting K-wires were inserted into the tibial or femoral medullar canal of the animals [2,21,23,24,25,30,33,35,38,39,40,41,42]. One weakness of these previous studies is that K-wires were not subject to the shear forces that arise during their drilling in clinical practice. These forces are significant, and, under normal circumstances, can be expected to remove or damage the drug-eluting layer during the drilling.

Here, we address the problem of overcoming shear forces in these types of external fixation implants by developing highly adhesive drug-eluting coatings. We report two types of highly adhesive coating that can resist the forces of drilling, maintain their structural integrity, and have drug-eluting capability. One group of coatings was based on synthetic epoxy co-polymers, and the other was based on a natural polysaccharide compound, chitosan. Dried coatings are capable of resisting strong shear forces during drilling; upon rehydration, the coatings swell, forming drug-eluting gels.

The first group of coatings was based on a novel class of cross-linkable amphiphilic copolymers developed and characterized by Luzinov et al. [44]. These polymers are “brush-like” molecules that are made from three different monomers: glycidyl methacrylate (GMA, which cross-links the entire polymeric network and provides for its mechanical strength), oligoethylene glycol methacrylate (OEGMA, which creates hydrophilic domains), and lauryl methacrylate (LMA, which creates hydrophobic domains). Varying the ratio of the monomers in the polymerization reaction provides a way to control the mechanical strength and drug-releasing properties of the polymer. These macromolecules have recently gained scientific attention because they can be covalently attached to solid surfaces and colloidal objects [44,45]. OEGMA demonstrates low cytotoxicity, does not trigger an immune response, provides water compatibility, and exhibits an antifouling effect [44]. We hypothesized that it would facilitate the incorporation of hydrophilic drugs into the polymer. LMA, on the other hand, is hydrophobic and serves as the domain for hydrophobic drugs. It tunes the hydrophilic/hydrophobic balance of the resulting molecular brush, and its low glass transition temperature assists in the chain movement freedom of the brush during thermal annealing [44]. GMA provides epoxy groups that can react with nucleophilic groups (such as hydroxyl) on the activated surface [44]. For this reason, the surfaces of the K-wires in this study were activated by plasma ion bombardment prior to coating, followed by hydration to create hydroxyl groups on the wire surface [46,47]. The studied polymers have previously exhibited low cytotoxicity to osteoblasts, demonstrating moderate attachment to the polymer-coated substrates [44].

Additionally, we investigated chitosan as a “green” alternative to synthetic coatings. Chitosan is already used in numerous applications, including agriculture, food preservation, wastewater treatment, medicine, cosmetics, fisheries, packaging, and the chemical industry [48,49,50]. Recently, chitosan has been investigated as a material for tissue engineering scaffolds, wound healing enhancers, and drug delivery matrices. It exhibits in situ gelling, mucoadhesion, pH sensitivity, hydrophilic characteristics, and permeation enhancing effects, facilitating the development of chitosan drug carriers, which can be hydrogels, films, fibers, microspheres, nanoparticles, tablets, beads, etc. [51,52]. For example, Tang et al. investigated chitosan films loaded with ibuprofen for oral mucosal delivery applications [53]. Chitosan has also been researched as part of the composite material for drug delivery applications. Huo et al. prepared the chitosan-microcapsules/starch blend film-based drug delivery device for antofloxacin with the pH-dependent release [54].

## 2. Results and Discussion

### 2.1. PGMA-Based Coatings

#### 2.1.1. Scanning Electron Microscopy

**Figure 1:**
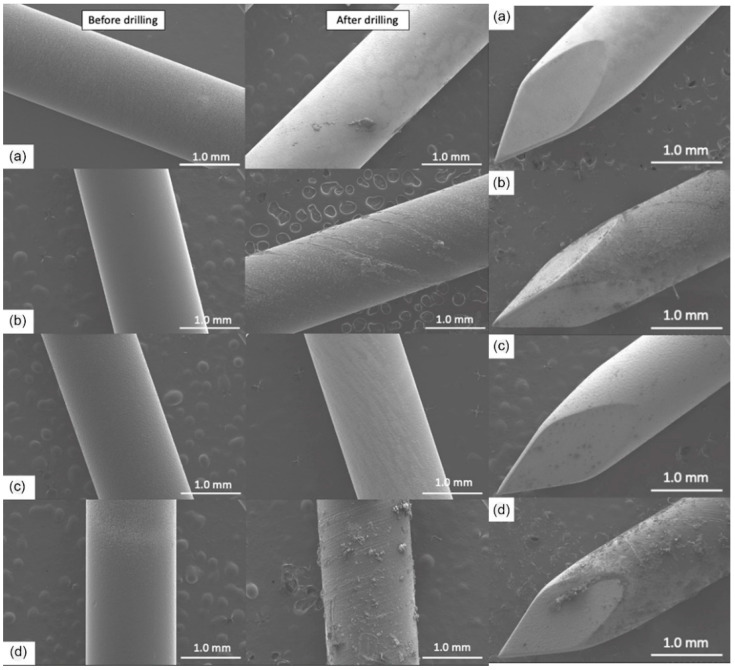
SEM images of gentamicin-loaded polymeric coatings. **Left panel**—before drilling; **middle panel**—after the drilling; **right panel**—tips after the drilling. (a) GS (5%). (b) P(G25-O70-L5) + GS (5%). (c) Gentamicin docusate-loaded PLA coating PLA + GD (5%). (d) P(G25-O50-L25) + GD (5%).

The coated K-wires were evaluated using SEM before and after the drilling (Figure 1). All polymer coatings were uniform and smooth prior to drilling (left panels). However, plain GS and PLA + GS coatings were largely removed by the drilling process (Figure 1A,C), as evidenced by the light color of the exposed metal surface. Gentamicin docusate (GD), P(GMA_25_-LMA_75_) + GD, and P(GMA_25_-OEGMA_70_-LMA_5_) + GD coatings were also essentially removed by drilling (images not shown). At the same time, P(GMA_25_-OEGMA_50_-LMA_25_) + GD and P(GMA_25_-OEGMA_70_-LMA_5_) + GS were still present after drilling (Figure 1B,D). The drilling-induced spiral striations can be seen on these coatings. The tips of the K-wires experienced the greatest stress of all the implant areas, and the coatings were sheared off from there more significantly than from the other drilled implant areas (right panel in Figure 1).

#### 2.1.2. Drug Loading

**Figure 2:**
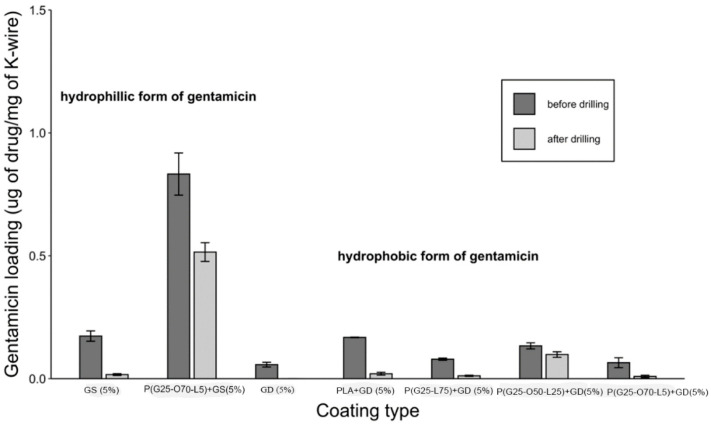
Gentamicin loading in PGMA-based gels before and after the drilling.

The drug loading efficiency was determined by measuring the total content of antibiotics in the drug-eluting gels. Additionally, the drug loading yield was verified during the measurements of the drug release. As can be seen in Figure 2, the K-wires that were coated with GS-containing gels had significantly higher gentamicin content than their GD-coated counterparts before drilling (*n* = 15, *p* < 0.05). However, most samples lost over 90% of the drug after drilling. Plain drug coatings had very low amounts of the drug left on their surfaces after drilling that are not visible on the plot (Figure 2). Only P(GMA_25_-OEGMA_70_-LMA_5_) + GS-coated and P(GMA_25_-OEGMA_50_-LMA_25_) + GD-coated K-wires retained over 61% and 74% of initially loaded gentamicin, respectively, in good agreement with the SEM results. For this reason, the further experiments focused specifically on these two coatings.

#### 2.1.3. Film Thickness and Wettability

The film thickness was measured by profiling the surface in terms of the height difference between the base silicon wafer surface and the coating surface. For P(GMA_25_-OEGMA_50_-LMA_25_) + GD coating, this height difference was found to be 5.3 ± 0.5 μm, while for P(GMA_25_-OEGMA_70_-LMA_5_) + GS, it was 8.7 ± 0.8 μm. In general, thinner coatings are preferable for orthopedic implants because they have a smaller chance of mechanical failure and promote bone integration more effectively than their thicker counterparts [55]. The contact angle for P(GMA_25_-OEGMA_50_-LMA_25_) + GD coating was 85 ± 6 degrees (*n* = 5), indicating a hydrophobic surface, whereas the contact angle for P(GMA_25_-OEGMA_70_-LMA_5_) + GS was 41 ± 11 degrees, indicating a hydrophilic surface.

#### 2.1.4. Drug Release

**Figure 3:**
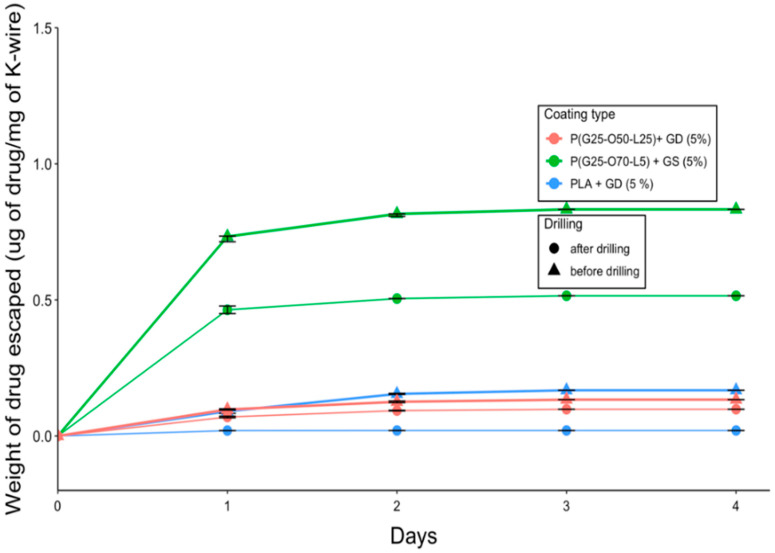
Gentamicin release from the PGMA-based gels on K-wires.

The PLA + GD coating demonstrated the slowest release before the drilling, with nearly 50% of the drug remaining in the coating after day 1, but after the drilling, there was virtually no drug left on the K-wire (Figure 3). A burst release of gentamicin was observed for all other coatings, and the release kinetics were similar for the samples both before and after drilling (*n* = 15, *p* > 0.05).

#### 2.1.5. Activity against Planktonic *S. aureus*

**Figure 4:**
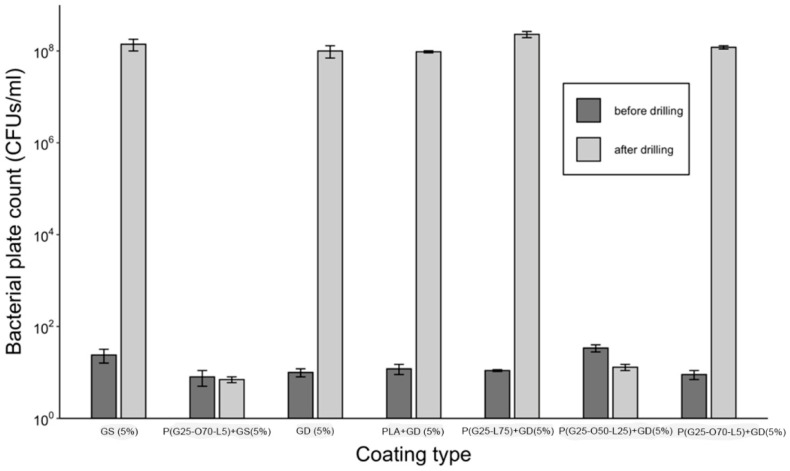
Antimicrobial efficacy against planktonic *S. aureus* (10^5^ CFUs/cm^2^ of coating challenge) in gentamicin-loaded gels.

A comparison of bacterial growth in the presence of PGMA-based coatings on K-wires in liquid broth before and after the drilling is shown in Figure 4. While all the coatings were able to eliminate 10^5^ CFUs before the drilling, only the P(GMA_25_-OEGMA_50_-LMA_25_) + GD- and P(GMA_25_-OEGMA_70_-LMA_5_) + GS-coated K-wires retained antimicrobial activity after the drilling (Figure 4). None of the other gentamicin-loaded coatings showed any antimicrobial activity after drilling.

#### 2.1.6. Zone of Inhibition

**Figure 5:**
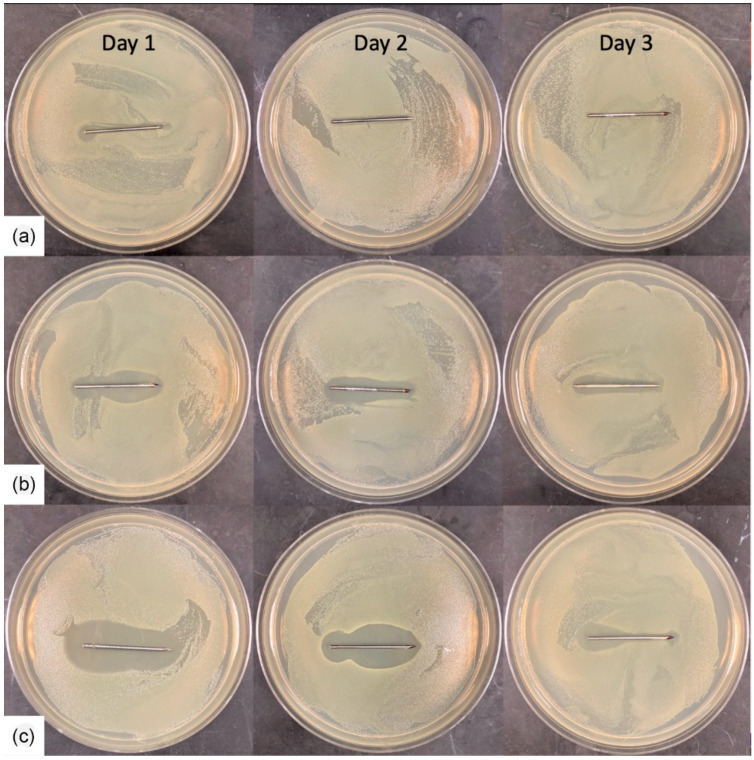
Inhibition zones of the coated K-wires after drilling. Trocar tips are shown on the right side. A newly seeded petri dish was used every day. (**a**) PLA + GD (5%). (**b**) P(G_25_-O_50_-L_25_) + GD (5%). (**c**) P(G_25_-O_70_-L_5_) + GS (5%).

The zone of inhibition experiment was only performed on the coated K-wires after the drilling (Figure 5). The clear zone around the K-wires represents the inhibition area where the CFUs were not able to grow. The PLA + GD-coated K-wires showed only a small zone at the base of the K-wire on day 1 and no zone around them for the following days (Figure 5A). The P(GMA_25_-OEGMA_70_-LMA_5_) + GS-coated K-wire showed a narrowing of the zone towards the trocar tip on day 3, and the P(GMA_25_-OEGMA_50_-LMA_25_) + GD-coated K-wire had clear uniform zones for 3 days, which indicates uniform coating, as is consistent with the SEM observations (*n* = 5) (Figure 5B,C).

### 2.2. Chitosan Coatings

#### 2.2.1. Scanning Electron Microscopy

**Figure 6:**
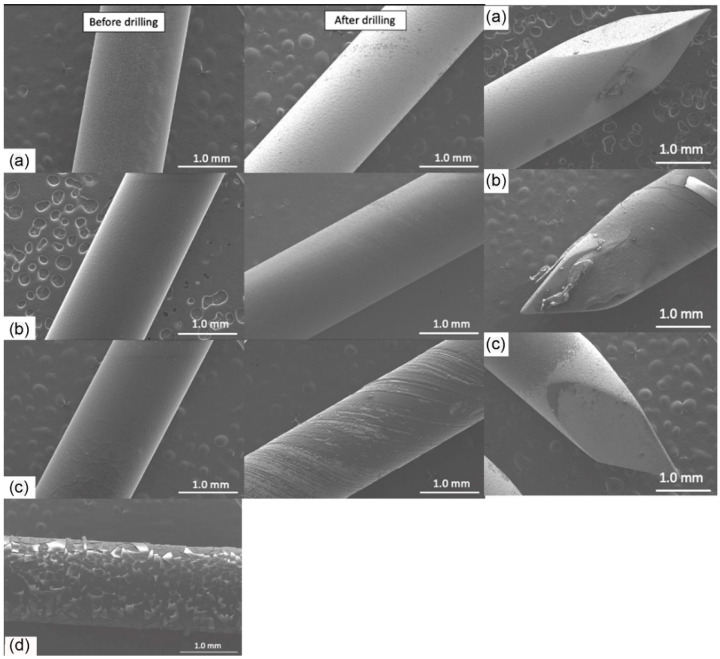
SEM images of the vancomycin-loaded chitosan coatings. **Left panel**—before drilling; **middle panel**—after the drilling; **right panel**—tips after the drilling. (a) VH (5%). (b) Chi Ace + VH (5%). (c) Chi Lac + VH (5%). (d) Chi Ace + VH (10%) before drilling.

The plain vancomycin hydrochloride (VH) coating was essentially removed by drilling (Figure 6). The Chi Lac + VH coating was partially removed by drilling, whereas the Chi Ace + VH (5%) coating demonstrated better adhesiveness and remained largely intact after drilling (Figure 6B,C). Chi Ace + VH (2.5%) was also able to retain some coating after drilling (images not shown), whereas Chi Ace + VH (10%) showed severe cracking before drilling, which is why it was excluded from further tests (Figure 6D). As mentioned above, the tips are subject to the highest shear forces. In the case of chitosan-coated wires after drilling, the coating was present only on the tips with chitosan acetate +5% VH (right panel in Figure 6).

#### 2.2.2. Drug Loading

**Figure 7:**
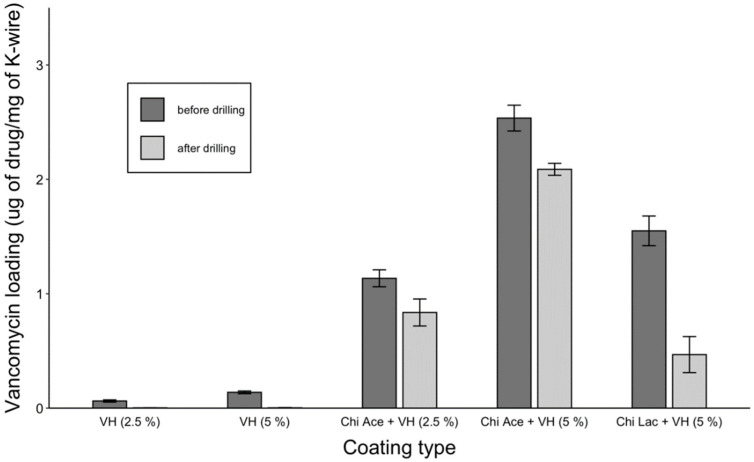
Vancomycin loading in chitosan gels on K-wires before and after drilling.

Increasing the vancomycin coating concentration led to an increased VH content on the K-wires compared to free vancomycin, and the chitosan acetate gels performed better than the chitosan lactate gels (Figure 7). A large standard deviation of the Chi Lac + VH coating after drilling indicated non-uniformity of the layer after the drilling, which is consistent with the SEM images. Chi Ace + VH (5%) retained 83% of its loaded drug after drilling. Plain drug coatings had minuscule amounts of the drug after drilling, which is not visible in the plot (Figure 7).

#### 2.2.3. Film Thickness and Wettability

Only Chi Ace + VH (5%) gels were tested in these studies because they showed superior properties to Chi Lac + VH gels and plain VH coatings in previous experiments. The coating thickness was measured to be around 7.5 ± 0.6 μm (*n* = 5). Contact angle goniometry revealed an angle of 62 ± 4 degrees.

#### 2.2.4. Drug Release

**Figure 8:**
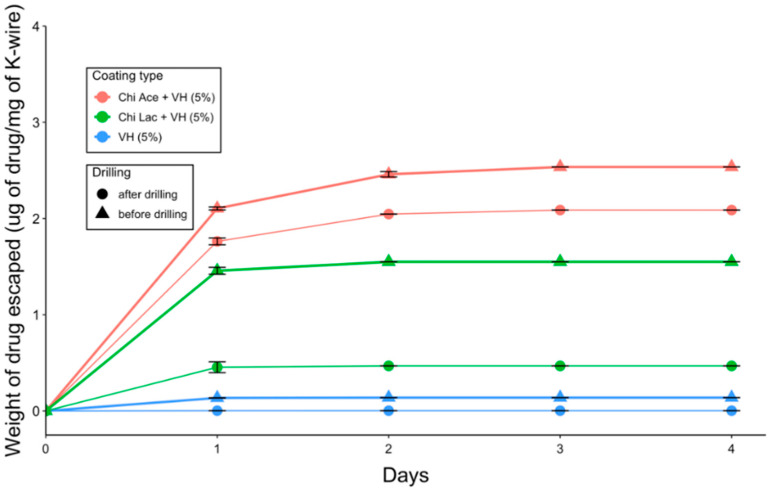
Vancomycin release from the coatings on the K-wires.

VH-coated K-wires also demonstrated burst releases of the drug into PBS (Figure 8). For Chi Ace + VH-coated K-wires, this occurred slightly more slowly than with the Chi Lac + VH-coated K-wires, but 85% of the drug was released within the first 24 h (*n* = 15). Drilling did not affect the release kinetics of vancomycin from any of the tested coatings (Figure 8).

#### 2.2.5. Activity against Planktonic *S. aureus*

**Figure 9:**
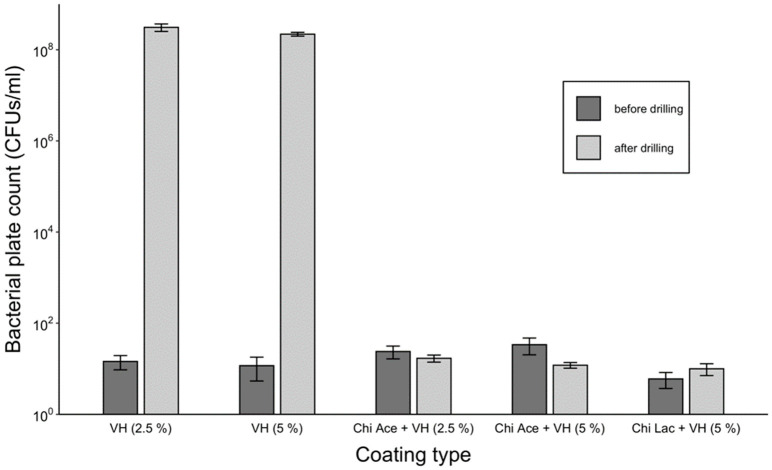
Antimicrobial efficacy against planktonic *S. aureus* (10^5^ CFUs/cm^2^ of coating) for vancomycin-loaded coatings.

Plain VH-coated K-wires were able to prevent the growth of bacteria before drilling, but failed to accomplish this after drilling (Figure 9). Chi Ace + VH and Chi Lac + VH coatings exhibited bactericidal properties before and after drilling, which is consistent with our previous observations.

#### 2.2.6. Zone of Inhibition

**Figure 10:**
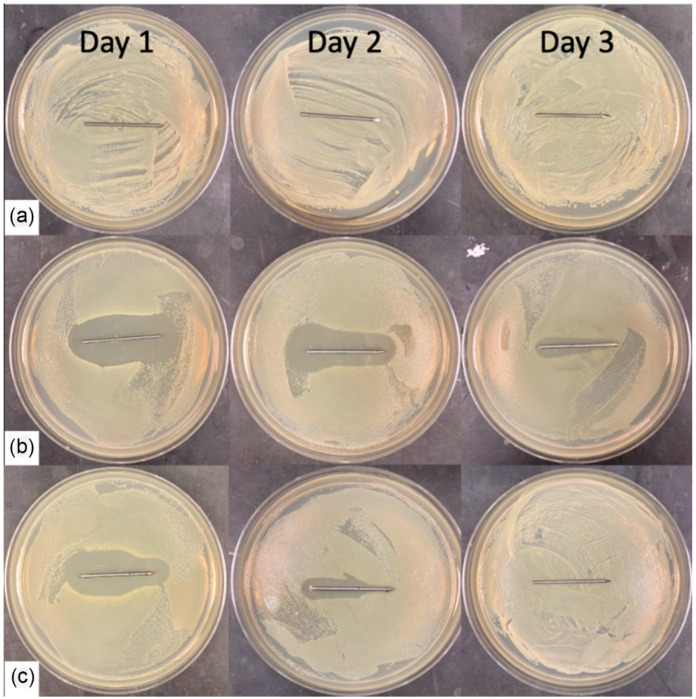
Inhibition zones of the coated K-wires after drilling. (**a**) VH (5%). (**b**) Chi Ace + VH (5%). (**c**) Chi Lac + VH (5%).

Figure 10 shows the VH-coated K-wires after drilling. Each K-wire was repositioned onto a newly seeded plate after 24 h or up to 72 h. The plain VH-coated wires had no zones around them, indicating total drug loss after drilling. Following the trend observed in the other experiments, Chi Ace + VH-coated K-wires performed better than Chi Lac + VH-coated K-wires, while Chi Lac + VH had significantly smaller inhibition zones that disappeared by day 3, and Chi Ace + VH had a clearly present zone on day 3 (*n* = 5, *p* < 0.05).

#### 2.2.7. Anti-Biofilm Activity of the Selected Coatings

**Figure 11:**
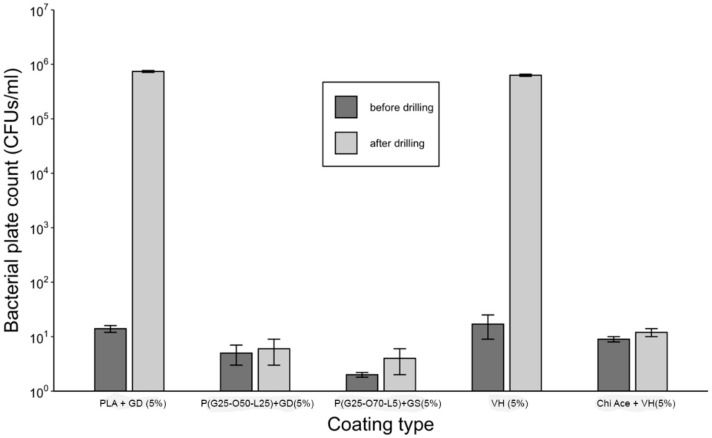
Anti-biofilm activity of the selected gels before and after the drilling.

This study was conducted on the best-performing coatings together with PLA + GD control and plain VH (5%) control coatings. The results seem to be in good correlation with the studies on planktonic bacteria, where P(G_25_-O_50_-L_25_) + GD, P(G_25_-O_70_-L_5_) +GS, and Chi Ace + VH (5%) gels were all able to prevent the growth of biofilms after drilling (Figure 11).

### 2.3. Discussion

The main complications of the external fixation devices are pin site loosening and pin site infection [5]. To complicate the matter further, both contribute to each other’s development: loosening may act as a portal for infection, and vice versa, inflammation caused by infection prevents proper osteointegration [5].

*Staphylococcus aureus* is the major pathogen associated with orthopedic implant infections, as nearly 60% of them are caused by this pathogen [56,57]. It possesses several cell-surface adhesion molecules that facilitate its binding to the metal and bone matrix [22]. The “race for the surface” concept states that the key for preventing implant-related infections is the host tissue integration occurring prior to bacterial attachment and subsequent biofilm formation [58]. Not only do biofilms provide a continuous release of bacteria into the surrounding tissues, but they are also 100–1000 times more resistant than the freely floating bacteria in terms of the antibiotic concentration required to kill them [29,58].

Polymeric coatings on K-wires have been a commonly proposed method to prevent pin site infections, as they can provide a local delivery of bioactive molecules and reduce bacterial attachment to the wire surface [2,21,23,24,25,30,33,34,39,40,41,42,43,57,59,60]. Miller et al. showed that the polycaprolactone (PCL) film on K-wires loaded with vancomycin prevented biofilm formation on the wire surface [21]. Likewise, Bernthal et al. developed a branched poly(ethylene glycol)-poly(propylene sulfide) (PEG-PPS) polymer coating designed to deliver antibiotics [39].

Figure 2 compares gentamicin loading on the coated K-wires before and after the drilling. Expectedly, all the polymer (whether PGMA-based or PLA)-gentamicin-coated K-wires had significantly more gentamicin weight on the surface than the plain gentamicin-coated K-wires before the drilling (Ps < 0.05). Nevertheless, the amounts of antibiotic present on plain gentamicin-coated wires were still sufficient to neutralize 5 × 10^5^ CFUs challenge before drilling as can be seen in Figure 4. However, only the P(GMA_25_-OEGMA_50_-LMA_25_)-GD- and P(GMA_25_-OEGMA_70_-LMA_5_)-GS-coated K-wires retained their antimicrobial properties after the drilling (Figure 4 and Figure 5). SEM images confirm that out of all of the PGMA-based coatings, only these two were still present on the wire surface after drilling (Figure 1). Both of the formulations that did not work, i.e., the P(GMA_25_-OEGMA_70_-LMA_5_)-GD and the P(G_25_-L_75_)-GD coatings, were almost completely scraped off after drilling. This indicates that the brush macromolecules may form different colloidal structures in the solution based on their constituents’ ratio, which may affect the adherence strength of the resulting gels. The amount of co-polymer composition also affected gentamicin loading, with hydrophilic polymer/hydrophilic drug P(GMA_25_-OEGMA_70_-LMA_5_)-GS formulation showing a loading yield 3–4 times higher than all other formulations (*p* < 0.05) (Figure 2). OEGMA sidechains are much longer than the LMA ones (M_n_ = 950 Da and M_n_ = 254 Da); therefore, hydrophilic domains may prevail in the overall structure of the gel.

As for the PLA-gentamicin-coated K-wires, only few remnants of the layer were observed on the surface after the drilling of what used to be a uniform coating prior to the drilling (Figure 1). There are many in vivo studies in the literature that have successfully tested PLA-gentamicin coatings on the K-wires [23,24,30,33,37,43,60]. However, in all of the reviewed studies, PLA-gentamicin-coated K-wires were either inserted into the femoral/tibial medullar canal [23,24,30,33,43,60] or passed through the predrilled holes [37]. In our study, the K-wires were drilled with constant forward power into the synthetic cortical bone model. This type of application generates much greater friction and heating; it can perhaps explain the difference in the results between our study and previous studies.

Virtually no difference in terms of gentamicin release kinetics was observed across all of the gentamicin-coated K-wires (Figure 3). According to our results, cross-linking of the polymer matrix did not prevent the burst escape of gentamicin from the gel coating into PBS. The conversion of gentamicin into its hydrophobic form (GS to GD) slightly slowed down the gentamicin release rate, but not in a statistically significant manner (Figure 3). One drawback of this standard in vitro testing is that it does not quite mimic the clinical situation where the K-wires are inserted in the hard bone rather than being shaken in the liquid. We suspect that the ionic strength of PBS is great enough to separate the gentamicin molecule from its hydrophobic counter-ion, docusate, and as the coating swells in PBS, gentamicin immediately escapes. We therefore decided to perform the zone of inhibition experiment to observe whether the release of gentamicin into the agar gel would be slowed down compared to its release into the liquid under shaking, as it would mimic the real-life scenario somewhat more closely. Indeed, a clear zone around the P(GMA_25_-OEGMA_50_-LMA_25_)-GD-coated K-wire was observed up until day 3 of the study, indicating that gentamicin was still being released at that time (Figure 5). The zone around the P(GMA_25_-OEGMA_70_-LMA_5_)-GS-coated K-wire appeared to be larger than the zone around the P(GMA_25_-OEGMA_50_-LMA_25_)-GD-coated K-wire, which is consistent with the higher drug loading used for the former formulation (Figure 2).

Additionally, we investigated chitosan as a “green” alternative to the synthetic PGMA-based gels. Chitosan is a natural polysaccharide that is known for producing mechanically strong films and displaying anti-bacterial properties [5,32,48]. The latter is attributed to the presence of positively charged amine groups on the chitosan backbone, which bind negatively charged bacterial membranes and induce membrane leakage [32]. In our study, however, plain chitosan acetate- and plain chitosan lactate-coated K-wires were not efficient against 5 × 10^5^ CFU *S. aureus* challenges even before drilling. In cases of drug-loaded chitosan coatings, chitosan acetate gels had significantly higher VH loads than chitosan lactate gels (*p* < 0.05). In general, using acetates for biomaterials is more effective than lactates, since the latter may cause lactic acidosis, which may lead to a decreased pH at the injury site [61].

All of the chitosan acetate-VH-coated K-wires had significantly more vancomycin loaded onto the wire surface than the corresponding plain VH-coated K-wires (*p* < 0.05) (Figure 7). Before drilling, all chitosan acetate-VH coated K-wires and plain VH-coated K-wires were efficient against 5 × 10^5^ CFUs of planktonic *S. aureus* inoculum (Figure 9). Three chitosan acetate-VH gels (with 2.5, 5, and 10% *w*/*w* VH in the coating solutions) retained antimicrobial efficacy after drilling (Figure 9). These findings correlated with the presence of the coating in these samples on SEM images after drilling (Figure 6).

Similarly to gentamicin sulfate, vancomycin hydrochloride is also an extremely hydrophilic drug and showed burst release from all the vancomycin-coated K-wires (Figure 8). It seems that chitosan acetate slowed down the escape of vancomycin into PBS, but no statistically significant difference was observed between the drug release rates for chitosan acetate-VH-coated K-wire and plain VH-coated K-wires. In the zone of inhibition experiment, however, the chitosan acetate-VH-coated K-wires had clear zones around them up until day 3, whereas for the plain VH-coated K-wires, only the 5 *w*/*w*% VH-coated K-wire had a small zone around it even on day 1 (Figure 10). The chitosan acetate gels performed better than the chitosan lactate gels in our zone of inhibition experiment.

In this study, gentamicin and vancomycin were used to model small antimicrobial agents that can be loaded into drug-eluting gels. Both antibiotics are commonly used to treat orthopedic infections [25,39]. The advantages of these drugs include heat degradation resistance, allowing us to thermally anneal PGMA-based polymer–gentamicin coatings at 120 °C, and these drugs do not interfere with the local bone healing [3]. However, local delivery of antibiotics can cause in situ drug resistance of the bacteria at the wound site [20]. This phenomenon sparked a search for alternative bactericidal substances such as bacteriophages [62] and natural fatty acids [20]. Our developed coatings should work with these other antimicrobial agents if they are co-soluble with the PGMA-based polymers or chitosan. We did not test chitosan–gentamicin coating solutions because both GS and GD have counter ions that cannot be co-solubilized with chitosan. The PGMA-based polymers with high OEGMA content are much less selective in that regard; these polymers can be dissolved in both aqueous and organic solvents, and therefore can be co-solubilized with both hydrophilic and hydrophobic drugs, as was demonstrated in our study.

## 3. Conclusions

In summary, we developed and characterized two novel coatings for external fixation devices: a synthetic PGMA-based coating and a natural chitosan coating. Both coatings turned out to be highly adhesive, and both were able to withstand the shear stress of drilling into hard tissue, unlike the PLA drug-loaded and plain drug coatings. Further research should be aimed towards understanding how the composition of the brush macromolecules affects the film’s properties, such as binding yield and adherence strength. Moreover, our methodology can be improved by making the necessary changes to prolong the burst release of the drugs to last multiple days. As the K-wires stay inserted for multiple weeks, prolonged release of the drug is favored. Early release of the drug is important during that period, since, besides the K-wire itself, bacteria can be introduced to soft subdermal and bone tissues at the wound site even before the surgery takes place in cases of open fractures. Thus, we conclude that our developed coatings could be promising for application on K-wires to reduce the incidence of pin site infections.

## 4. Materials and Methods

### 4.1. Materials

Stainless steel K-wires (1.6 mm in diameter, SKU 40-1569) were obtained from Sklar (West Chester, PA, USA). Vancomycin hydrochloride was purchased from Alfa Aesar (Tewksbury, MA, USA). Gentamicin sulfate, glycidyl methacrylate (GMA), oligo-ethylene-glycol methacrylate (OEGMA), lauryl methacrylate, methyl ethyl ketone (MEK), glacial acetic acid, AIBN initiator, inhibitor removers, sodium docusate, polylactic acid (P1691), calcium chloride, potassium chloride, sodium acetate, dichloromethane (DCM), *o*-phtalaldehyde (OPA), and phosphate buffer saline (PBS) were purchased from Millipore Sigma (St. Louis, MO, USA). A synthetic human femur bone model (#3414) was purchased from Sawbones (Vashon Island, WA, USA). Chitosan and chitosan lactate were provided as a gift by Tidal Vision USA (Bellingham, WA, USA). Difco™ Tryptic Soy Agar and BBL™ Trypticase™ Soy Broth (TSB) were purchased from BD (Franklin Lakes, NJ, USA). *Staphylococcus aureus* (ATCC 33591, KWIK-STIK^TM^ Plus) was obtained from Microbiologics (Saint Cloud, MN, USA).

### 4.2. Synthesis of PGMA-Based Polymers

**Figure 12:**
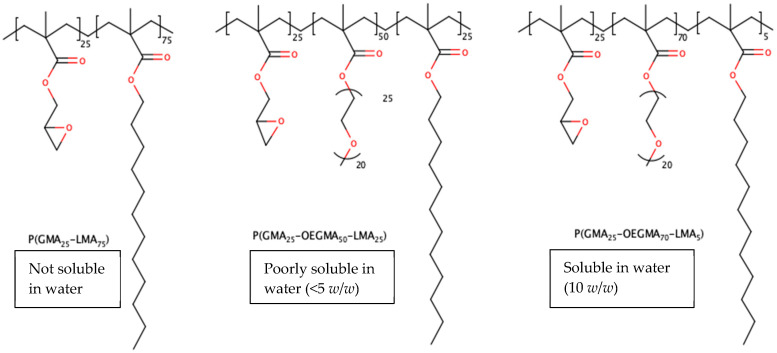
Overview of the studied polymers. Solubility in water was reported prior to crosslinking. After the crosslinking at 120 °C, the polymeric gels were not soluble, but were swellable in water.

The polymers were synthesized in accordance with the technology developed by Luzinov et al. [42]. Solution radical copolymerization was used to synthesize three different polymers based on monomeric molar feeds: P(GMA_25_-OEGMA_50_-LMA_25_), P(GMA_25_-OEGMA_70_-LMA_5_), and P(GMA_25_-LMA_75_), where the subscripts designate the molar feed percent. Their average molecular weights, reactivity ratios, thermal behavior, and other properties were characterized previously [44]. Briefly, the monomers—GMA, OEGMA, and LMA—were dissolved in MEK together with inhibitor removers, which were present in the monomer mass to prevent spontaneous polymerization. Afterward, the monomer solutions were added together and diluted further in MEK. Initiator AIBN was added to the mixture, followed by purging the solution with nitrogen for 30 min to remove oxygen from the sealed flask. Finally, the reaction mixture was heated to 50 °C for 1.5 h, then terminated by exposure to the ambient atmosphere. Both the unreacted monomers and initiator were removed by the three cycles of centrifugation/re-solubilization in diethyl ether, in which they are soluble, but the co-polymers are not. The three polymers were dissolved in MEK; the weight concentration was determined by solvent evaporation. P(GMA_25_-OEGMA_70_-LMA_5_), being the only studied synthetic water-soluble polymer in relevance to coating solution concentrations, was also dissolved in DI water (Figure 12).

### 4.3. Synthesis of Gentamicin Docusate (GD)

Since both gentamicin sulfate (GS) and vancomycin hydrochloride (VH) are extremely hydrophilic, they could not be co-solubilized with the polymers in MEK. The hydrophobic ion pairing (HIP) method was employed to convert gentamicin to its hydrophobic form [62]. In short, equal volume proportions of GS in aqueous buffer (10 mM calcium chloride, 10 mM potassium chloride, 10 mM sodium acetate, pH 5.0) and sodium docusate (NaAOT) in dichloromethane (DCM) were mixed vigorously for 3 h. The molar ratio of GS to NaAOT was 5:1, as gentamicin has five ionizable amine groups. After 3 h, the organic phase was separated by centrifuging, then dried under nitrogen flow. The resulting gentamicin docusate (GD) was collected as a waxy solid and redissolved in MEK. The fraction of gentamicin that remained in the aqueous phase was quantified using o-phtalaldehyde (OPA) reagent assay adapted from the literature [63]. In all attempted procedures, the yield of gentamicin in organic phase was at least 90% of the initial feed (20 mg/mL).

### 4.4. Preparation of Chitosan Solutions

Chitosan is generally not soluble in water due to strong inter- and intramolecular bonding. However, it can be dissolved under acidic conditions. We dissolved commercially obtained chitosan in dilute acetic acid (1% *w*/*w*) to produce chitosan acetate (Chi Ace) solutions. Chitosan lactate (Chi Lac) is a water-soluble form of chitosan, and it was provided by the manufacturer in the form of flakes, which were dissolved in DI water.

### 4.5. Preparation of Coating Solutions

#### 4.5.1. PGMA-Based Coating Solutions

Individual polymers were co-dissolved with GD in MEK to produce final concentrations of 10% *w*/*w* polymer and 5% *w*/*w* drug. A polylactic acid (PLA) drug-eluting coating was used as the “gold standard” control. PLA was co-dissolved with GD in MEK with the same concentrations. P(GMA_25_-OEGMA_70_-LMA_5_) was co-dissolved with GS in DI water using the same 10%/5% final coating concentrations (Table 1).

#### 4.5.2. Chitosan Coating Solutions

Gentamicin sulfate could not be used with chitosan because sulfate precipitates chitosan from the aqueous solution. Instead, vancomycin hydrochloride (VH) was co-dissolved with chitosan acetate (Chi Ace) and chitosan lactate (Chi Lac). Chi Lac/VH coating solutions had 2.7% *w*/*w* of Chi Lac and 5% *w*/*w* of the drug, whereas Chi Ace/VH coating solutions had 1.35% *w*/*w* of Chi Ace and variable weights of the drug: 2.5%, 5%, and 10%, all in *w*/*w* (Table 1).

#### 4.5.3. Coating Procedure

Prior to coating, all K-wires were sandblasted with glass beads as abrasive blasting media (6ZC13, Grainger, Lake Forest, IL, USA); cut into 10 cm pieces; and washed in an ultrasonic bath in 95% acetone, followed by 95% propanol and 99% ethanol for 10 min each. After air-drying, K-wires were subjected to plasma treatment to activate the surfaces in accordance with the literature [46]. Briefly, the K-wires were kept under the linear beam source while the plasmas were struck with radio frequency (RF) power at 27 MHz for 10 min (PDC-002-HP, Harrick Plasma, Ithaca, NY, USA). For the synthetic polymer coatings, oxygen plasma was used to create anchoring hydroxyl radicals on the wire surface, whereas for the chitosan coatings, carbon dioxide plasma was used to produce carboxyl radicals on the wire surface that are able react with amine groups of chitosan. The K-wires were then immediately placed in the DI degassed water for surface radical hydration and quickly dip-coated in the corresponding polymer solution at a withdrawing speed of 80 mm/min using the D-3400 Dip-Coater (Mayer Feintechnik, Gottingen, Germany). Only half (~5 cm) of each K-wire was coated. Following air-drying, the PGMA-based coated K-wires were subjected to thermal annealing at 120 °C under an oxygen-free atmosphere to achieve epoxy ring-opening cross-linking for 8 h [44]. Finally, the K-wires were allowed to cool down to room temperature before their characterization.

### 4.6. Drilling into the Bone Model

**Figure 13:**
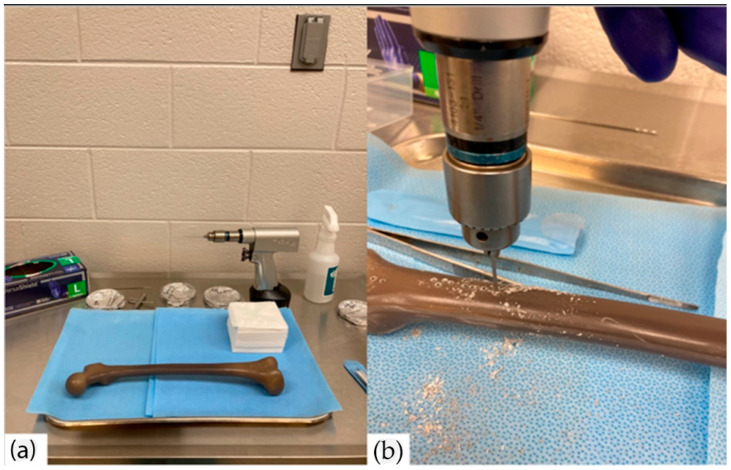
Drilling of K-wires into human femur-mimetic material. (**a**) Experimental setup of the operation. (**b**) Drilling was carried out at 90° angle under constant pressure.

Drilling of the coated K-wires was performed at the Godley Snell Research Facility (GSRF) at Clemson University (Figure 13). The K-wires were drilled using a surgical drill (Stryker Model 5) at 1200 RPM under constant pressure into the synthetic human femur bone model (#3414, Sawbones, Vashon Island, WA, USA). The K-wires were left drilled in for 1 min and then drilled out for further examination. For some studies, the properties of the coatings were compared before and after the drilling.

### 4.7. Scanning Electron Microscopy

Scanning electron microscopy images of the coated K-wires before and after drilling were obtained using an S4800 microscope (Hitachi, Tokyo, Japan). Qualitative assessment of the coating is possible due to the high contrast between the non-electroconductive polymeric layer and the highly-electroconductive metal surface.

### 4.8. Drug Loading of PGMA-Based Copolymer and Chitosan Gels

For the drug loading measurements, the coated K-wires were immersed in DMSO under vigorous shaking for 2 h at 50 °C. This procedure desorbs and dissolves both the coating and the encapsulated drugs. Gentamicin sulfate and gentamicin docusate were measured by the *ortho*-phtaldialdehyde (OPA) reagent assay protocol [63]. Briefly, (OPA) reacts with amine groups in gentamicin to create a fluorescent product with 340 nm/455 nm excitation/emission. Vancomycin hydrochloride on chitosan-coated wires was measured in a similar fashion. Vancomycin was measured using UV-VIS spectroscopy at 284 nm in DMSO (Epoch microplate reader, Agilent, Santa Clara, CA, USA). Standard curves for all drug measurements were used as references for the concentrations. These measurements were performed before and after the drilling to evaluate the drug loss induced by drilling, and were standardized per unit length and unit weight of the K-wire.

### 4.9. Film Thickness and Wettability

The film thickness and surface energy (wettability) were assessed using a non-contact profilometer (VK-X3000, Keyence, Itasca, IL, USA) on the coated silicon wafers. The hydrophilicity of the coatings was assessed through water contact angle measurements using contact angle goniometry with 1.0 μL water drops (DSA30E, Kruss Scientific, Hamburg, Germany).

### 4.10. Sterilization

The K-wires were sterilized using an autoclave, as both gentamicin and vancomycin are able to withstand autoclaving conditions [64,65]. Sterilized K-wires were used for all in vitro experiments.

### 4.11. Drug Release from PGMA-Based Copolymer and Chitosan Gels

The coated 3.5 cm K-wire pieces were placed into 1 mL of phosphate buffer saline (PBS) at 37 °C under mild shaking. The solutions were replaced every 24 h, and the released drug was measured in those solutions using the OPA assay for gentamicin and UV-VIS spectroscopy at 284 nm for vancomycin, as described above.

### 4.12. Antimicrobial Efficacy of PGMA-Based Copolymer and Chitosan Coatings

For all bacterial studies, *Staphylococcus aureus* (ATCC 33591) was used, as this species is responsible for the vast majority of the implant-associated infections. All the bacterial cultures were used at the active mid-log phase of proliferation and in at least the third culture passage after the fridge/freezer.

### 4.13. Activity against Planktonic Bacteria

The coated K-wire pieces, 1 cm long, were placed individually into 1 mL of 10^5^ colony forming units (CFUs)/mL in liquid broth at 37 °C under mild shaking. After 18 h, 100 μL aliquots were taken from each suspension with the K-wire and plated onto tryptic soy agar in triplicate. The number of CFUs was calculated after 24 h of incubation of the plates.

### 4.14. Anti-Biofilm Activity

The biofilm formation could not be quantified by the commonly used crystal violet assay (CV) because CV also stains the polymeric coatings on the K-wire surface. Instead, a different method from the literature [66] was adapted for this study. To grow biofilms, K-wires were incubated at 10^5^ CFUs/mL in liquid broth at 37 °C under mild shaking for 48 h, followed by rinsing with PBS to remove loosely attached bacteria. After rinsing, the K-wires were subject to vigorous vortex-mixing for 30 s, followed by sonication in the ultrasonic water bath for 1 min to detach bacteria in biofilm. This “biofilm-desorbing” process was repeated two more times. Plate colony counting was then performed to determine the number of CFUs in the vortexed and sonicated suspension.

### 4.15. Zone of Inhibition

Drilled and coated K-wire pieces 3 cm long were placed individually on the 10^7^ CFUs-seeded tryptic soy agar plates. After 24 h, the radius of inhibition zone was measured using a ruler, and the K-wires were repositioned onto the new 10^7^ CFUs-seeded tryptic soy agar plates. This procedure was repeated up to day 3 of the experiment. This experiment allowed us to model the drug release conditions more accurately to real-life conditions than the standard test with a liquid medium.

### 4.16. Statistical Analysis

Comparison between the groups was performed using one-way ANOVA and Fisher’s least significant difference test. Differences were considered statistically significant at *p* < 0.05.

## Figures and Tables

**Table 1:** Summary of the implant-coating solutions used in the study.

Controls	PGMA-Based Coating Solutions	Chitosan Coating Solutions
Gentamicin sulfate (GS) 5% in H_2_O (plain drug control)	P(GMA_25_-OEGMA_70_-LMA_5_) 10% + GS 5% in H_2_O	Chitosan Lactate 2.7% + VH 5% in H_2_O
Gentamicin docusate (GD) 5% in MEK (plain drug control)	P(GMA_25_-LMA_75_) 10% + GD 5% in MEK	Chitosan Acetate 1.35% + VH 2.5% in H_2_O
Vancomycin Hydrochloride (VH) 5% in H_2_O (plain drug control)	P(GMA_25_-OEGMA_50_-LMA_25_) 10% + GD 5% in MEK	Chitosan Acetate 1.35% + VH 5% in H_2_O
Polylactic acid (PLA) 10% + GD 5% in MEK (commercial coating control)	P(GMA_25_-OEGMA_70_-LMA_5_) 10% + GD 5% in MEK	Chitosan Acetate 1.35% + VH 10% in H_2_O
